# Phytochemical and Biological Activity Studies on *Nasturtium officinale* (Watercress) Microshoot Cultures Grown in RITA^®^ Temporary Immersion Systems

**DOI:** 10.3390/molecules25225257

**Published:** 2020-11-11

**Authors:** Marta Klimek-Szczykutowicz, Michał Dziurka, Ivica Blažević, Azra Đulović, Sebastian Granica, Izabela Korona-Glowniak, Halina Ekiert, Agnieszka Szopa

**Affiliations:** 1 Chair and Department of Pharmaceutical Botany, Faculty of Pharmacy, Jagiellonian University, Medical College, Medyczna 9, 30-688 Kraków, Poland; marta.klimek-szczykutowicz@doctoral.uj.edu.pl (M.K.-S.); halina.ekiert@uj.edu.pl (H.E.); 2 Polish Academy of Sciences, The Franciszek Górski Institute of Plant Physiology, Niezapominajek 21, 30-239 Kraków, Poland; m.dziurka@ifr-pan.edu.pl; 3 Department of Organic Chemistry, Faculty of Chemistry and Technology, University of Split, Ruđera Boškovića 35, 21000 Split, Croatia; blazevic@ktf-split.hr (I.B.); azra@ktf-split.hr (A.Đ.); 4 Department of Pharmacognosy and Molecular Basis and Phytotherapy, Medical University of Warsaw, Banacha 1, 02-097 Warszawa, Poland; sgranica@wum.edu.pl; 5 Department of Pharmaceutical Microbiology, Faculty of Pharmacy, Medical University of Lublin, Chodźki 1, 20-093 Lublin, Poland; iza.glowniak@umlub.pl

**Keywords:** in vitro cultures, temporary immersion system (TIS), RITA^®^ bioreactor, glucosinolates, polyphenols, antioxidant potential, anti-inflammatory activity, antibacterial activity, antifungal activity

## Abstract

The main compounds in both extracts were gluconasturtiin, 4-methoxyglucobrassicin and rutoside, the amounts of which were, respectively, determined as 182.93, 58.86 and 23.24 mg/100 g dry weight (DW) in biomass extracts and 640.94, 23.47 and 7.20 mg/100 g DW in plant herb extracts. The antioxidant potential of all the studied extracts evaluated using CUPRAC (CUPric Reducing Antioxidant Activity), FRAP (Ferric Reducing Ability of Plasma), and DPPH (1,1-diphenyl-2-picrylhydrazyl) assays was comparable. The anti-inflammatory activity of the extracts was tested based on the inhibition of 15-lipoxygenase, cyclooxygenase-1, cyclooxygenase-2 (COX-2), and phospholipase A_2_. The results demonstrate significantly higher inhibition of COX-2 for in vitro cultured biomass compared with the herb extracts (75.4 and 41.1%, respectively). Moreover, all the studied extracts showed almost similar antibacterial and antifungal potential. Based on these findings, and due to the fact that the growth of in vitro microshoots is independent of environmental conditions and unaffected by environmental pollution, we propose that biomass that can be rapidly grown in RITA^®^ bioreactors can serve as an alternative source of bioactive compounds with valuable biological properties.

## 1. Introduction

Plant in vitro cultures are an important part of biotechnological studies, which aid in investigating the potential of obtained biomass to be used as a source of secondary metabolites obtained, for example, from rare and protected plant species. The culture biomass can thus be used in pharmaceutical, cosmetological, and agricultural industries. Furthermore, the large-scale plant in vitro cultures allow for producing those compounds with limited natural resources or resources threatened by overexploitation [1,2]. 

In order to scale up the biomass production and secondary metabolite production, the special bioreactors for plant propagation are applied in plant biotechnology [3]. Depending on the environment in the cultivation chamber, four types of bioreactors are distinguished in plant biotechnology, namely liquid-phase bioreactor, gas-phase bioreactor, hybrid bioreactor, and temporary immersion system (TIS) [3]. TIS enables the cultivation of plant in vitro cultures in submerged and nonsubmerged cycles. In this system, in vitro cultures are maintained by immersing in liquid medium without mechanical stress and with good gas exchange [1,2,3]. Several plant in vitro cultures have been cultured in the TIS bioreactors for optimizing the production of secondary metabolites. For example, compounds including xanthone, benzophenone, and bioflavonoids were obtained from *Cyclopia genistoides* [4], flavonoids and other phenolics from *Rosa rugosa* and *Castilleja tenuiflora* [5,6], galantamine from *Leucojum aestivum* [7], and camptothecin from *Camptotheca acuminata* [8].

*Nasturtium officinale* R. Br. (Robert Brown), known as watercress, is a perennial, aquatic, or semiaquatic plant species with creeping or floating stems, which colonizes gently flowing and shallow streams in its natural habitat. It belongs to Brassicaceae family and is classified by the International Union for Conservation of Nature (IUCN) in the Red List of Threatened Species, as a plant of least concern in Europe. This species has different statuses in European countries; for instance, in Austria and Sweden, it is considered as an endangered species, while in Poland it is partly endangered [9]. The monograph of the *N. officinale* herb is present in German Commission E Monographs (Phyto-Therapy) [10]. The European Food Safety Authority (EFSA) has classified *N. officinale* as a safe vegetable under the group “Leaf vegetables, herbs and edible flowers” [11]. The major compounds found in the *N. officinale* herb are glucosinolates (GSLs), isothiocyanates, polyphenols (flavonoids, phenolic acids, proanthocyanidins), terpenoids (including carotenoids), vitamins (B1, B2, B3, B6, E, C), and bioelements [12,13,14,15]. An analysis of different plant organs from *N. officinale* such as leaves, stem, flower, and root indicated the presence of phenylalanine derivatives (gluconasturtiin and glucotropaeolin), methionine derivatives (glucoiberin, 7-(methylsulfinyl)heptyl GSL, glucohirsutin), and tryptophan derivatives (glucobrassicin, 4-hydroxyglucobrassicin, 4-methoxyglucobrassicin). Among these, the important compound in *N. officinale* is gluconasturtiin, the content of which is over 10-fold higher than the other GSLs [15,16]. The presence of these compounds and their breakdown products—isothiocyanates—is mostly responsible for the observed antioxidant, anticancer, antibacterial, and anti-inflammatory effects of the *N. officinale* extracts [17,18,19,20]. 

Previous biotechnological studies on *N. officinale* in vitro cultures focused on micropropagation and analyzing the ability of the plant to regenerate from callus culture [21] as well as on its genetic transformation by *Agrobacterium rhizogenes* and the resulting impact on the production of GSLs [22,23]. In our latest studies, we optimized the condition under which microshoots can be grown in agar and agitated cultures. We chose Murashige and Skoog (MS) medium with 1 mg/L 6-benzyladenine (BA) and 1 mg/L 1-naphthaleneacetic acid (NAA) as the appropriate medium for the cultivation of *N. officinale*, and determined the optimal growth period as 20 days for agar cultures and 10 days for agitated cultures [24]. In addition, we investigated and confirmed the ability to bioaccumulate several bioelements, such as calcium, chromium, copper, iron, lithium, magnesium, selenium, and zinc, in the biomass of *N. officinale* from agitated cultures [25]. 

These analyses prompted us to conduct further works on *N. officinale* and study the production of secondary metabolites in in vitro cultures of this species and evaluate its biological activity in highly productive TIS bioreactors.

The aim of the present research was to establish *N. officinale* RITA^®^ TIS microshoot cultures and to estimate the GSLs and phenolic compounds profiles. The extracts obtained from biomass were also evaluated for their antioxidant, anti-inflammatory, antibacterial, and antifungal activities. The results were compared with those of the herb extract obtained from the aerial parts of the parent plant with flowers.

## 2. Results

### 2.1. Microshoots Appearance and Biomass Growth

**Figure 1:**
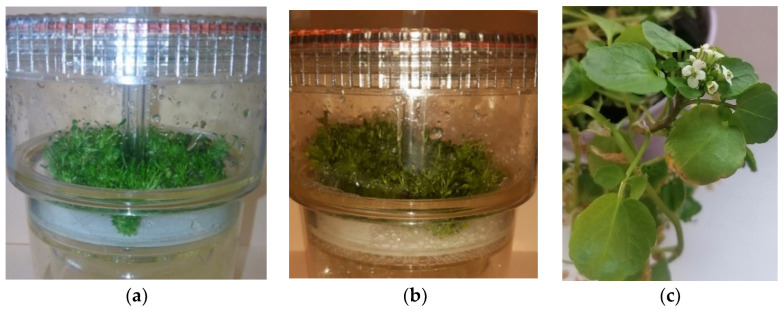
Microshoot cultures of *N. officinale* cultivated in bioreactor RITA^®^ TIS on MS medium containing 1 mg/L BA and 1 mg/L NAA after: 10 days of growth period (**a**); 20 days of growth period (**b**); and the herb of *N. officinale* (**c**).

The appearance of tested bioreactor cultures was dependent on the duration of growth period (Figure 1a,b). After 10 and 20 days, the number of shoots was large. The microshoots had dark green-colored leaves. A great increase in the fresh biomass was observed during the 10- and 20-day growth periods, while after 30 days, the biomass died and was not analyzed.

High growth index (Gi) values were obtained for the dry biomass. Depending on the culture duration, the increases in dry biomass, expressed by Gi, ranged from 16.54 (after 10 days) up to 31.71 (after 20 days). The Gi estimated for the cultures grown over 20 days was 1.9-fold higher than the increase recorded after 10 days. 

### 2.2. Individual and Total GSLs Contents

The results obtained from the analysis of individual and total GSLs contents are presented in Table 1 and Table 2. For in vitro cultures, the total GSLs content was found to be dependent on the duration of growth periods, i.e., 113.70 and 261.97 mg/100 g dried weight (DW) after 20 and 10 days, respectively (Table 1). In the case of *N. officinale* herb extract, the total GSLs content was 799.47 mg/100 g DW (Table 1). 

**Figure 2:**
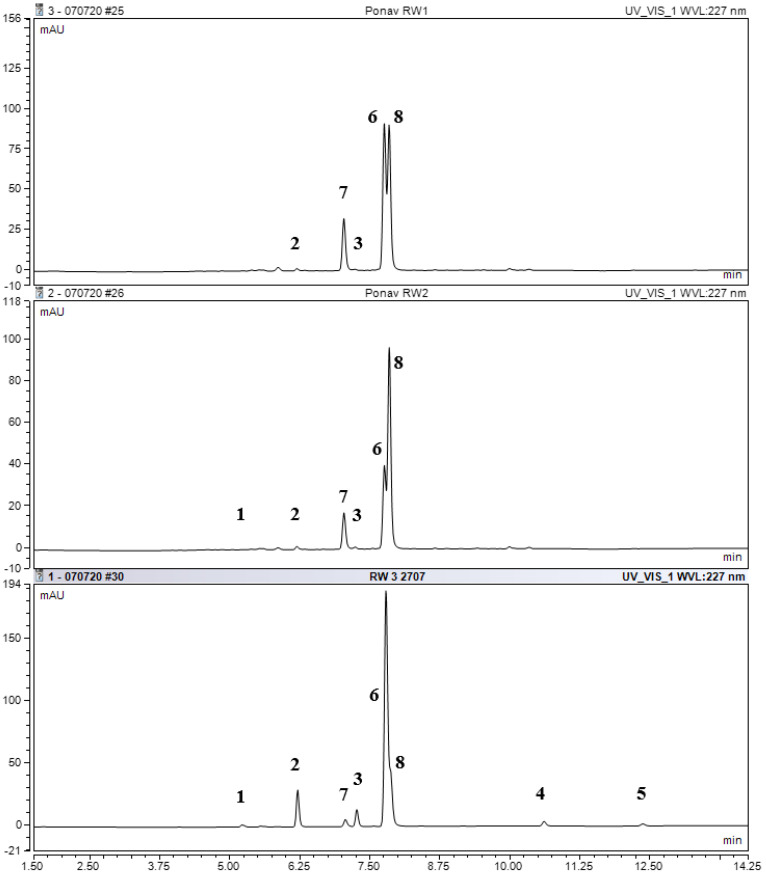
Chromatograms of desulfoglucosinolates (dGSLs): **RW1**—extract of 10-days bioreactors microshoot cultures, **RW2**—extract of 20-days bioreactors microshoot cultures, **RW3**—extract of *N. officinale* herb. For compound numbers, refer to Table 2.

After 10 and 20 days of growth, three main GSLs were found in the extracts of *N. officinale* bioreactor cultures using UHPLC-DAD-MS/MS: phenylalanine-derived GSLs (gluconasturtiin), followed by tryptophan-derived GSLs (glucobrassicin and 4-methoxyglucobrassicin) (Table 2, Figure 2). The highest content of gluconasturtiin (182.93 mg/100 g DW) was obtained in the bioreactor cultures after 10 days of growth, while its content decreased after 20 days (56.32 mg/100 g DW). The content of 4-methoxyglucobrassicin determined after 10 and 20 days was similar—58.86 and 49.76 mg/100 g DW, respectively. Glucobrassicin was accumulated in lower amounts in the tested extracts: 20.18 mg/100 g DW after 10 days and 7.62 mg/100 g DW after 20 days (Table 2). Additionally, methionine-derived GSLs (glucohirsutin and 7-(methylsulfinyl)heptyl GSL) were detected in the trace amounts in the biomass extracts.

The analysis of the extracts from the *N. officinale* herb revealed the presence of four main GSLs: phenylalanine-derived GSL (gluconasturtiin, 640.94 mg/100 g DW), methionine-derived GSLs (7-(methylsulfinyl)heptyl GSL, 92.27 mg/100 g DW and glucohirsutin, 42.79 mg/100 g DW), and tryptophan-derived GSLs (4-methoxyglucobrassicin, 23.47 mg/100 g DW). In addition, other GSLs were found in trace amounts, which included a methionine-derived compounds (glucohesperin, 7-(methylsulfanyl)heptyl GSL and 8-(methylsulfanyl)octyl GSL), and a tryptophan-derived compound (glucobrassicin) (Table 2, Figures S1 and S2).

### 2.3. Spectrophotometric Analysis of Total Polyphenol and Flavonoid Contents

The Folin–Ciocalteu (F-C) assay revealed the highest total polyphenol contents (3.74 mmol TE (trolox equivalent)/100 g DW) for the microshoots cultured over 10 days in bioreactor system, while the lower polyphenol contents (3.10 mmol TE/100 g DW) were detected for the biomass maintained for 20 days (Table 1). In the herb extracts, the total polyphenol content equaled 2.70 mmol TE/100 g DW (Table 1).

The total flavonoid content in the case of in vitro culture extracts was calculated as 0.95 and 1.64 mmol RE (rutoside equivalent)/100 g DW after 20 and 10 days of growth, respectively (Table 1). In the herb extract, the total flavonoid content was estimated at 1.89 mmol RE/100 g DW (Table 1).

### 2.4. Chromatographic Analysis of Polyphenol Contents

**Figure 3:**
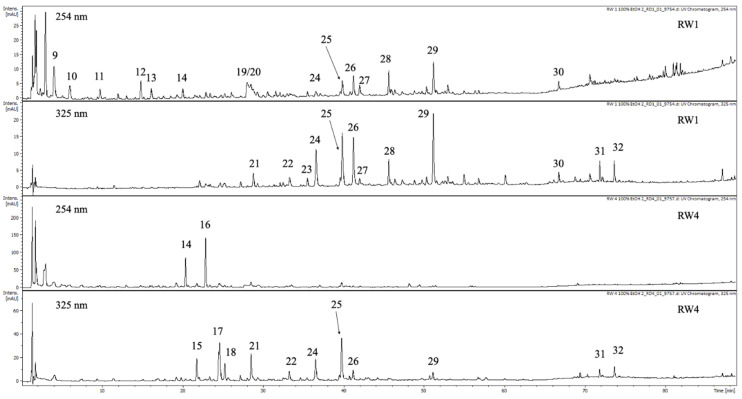
UHPLC-DAD-ESI-MS chromatogram of *N. officinale* extract from bioreactors cultures and the herb of the parent plant separation of the phenolic compounds (at 254 and 325 nm). For compound numbers, refer to Table 3. **RW1**—extract of *N. officinale* herb, **RW4**—extract of *N. officinale* bioreactors microshoot cultures.

The qualitative analysis of polyphenol compounds by the UHPLC-DAD-ESI-MS method showed the presence of *p*-coumaric (24) and ferulic acid (25) among phenolic acids in the in vitro biomass extracts. In addition, the derivatives of protocatechuic (14), caffeic (15, 18, 22), phenolic (derivatives) (17,21) and sinapic acids (29) were detected. Among the flavonoids, rutoside (26) was detected in these extracts. The presence of three undefined compounds (16, 31, 32) was also noted (Table 3, Figure 3).

In the extracts of the parent plant herb, the presence of four phenolic acids was confirmed: gallic (9), protocatechuic (10), *p*-coumaric (24) and ferulic (25) acids. In addition, the derivatives of protocatechuic (14), phenolic (21), caffeic (22, 23) and sinapic (29) acids were detected. Among flavonoids, rutoside (26), isoquercitrin (27) and kaempferol *O*-rhamnohexoside (28) were detected in the extracts of the *N. officinale* herb. Furthermore, six undefined compounds (11, 12, 13, 30, 31, 32) were detected in these extracts (Table 3). 

The amounts of four phenolic acids (*p*-coumaric, gallic, ferulic and protocatechuic acids) and three flavonoids (rutoside, isoquercitrin and kaempferol *O*-rhamnohexoside) detected using the UHPLC-DAD-ESI-MS analysis were estimated quantitatively by the HPLC-DAD method (Table 3). Quantitative analysis showed that the dominant compound in the extracts from in vitro cultures was ferulic acid; its content was dependent on the growth cycles and was estimated at 9.28 and 1.66 mg/100 g DW after 10 and 20 days of growth period, respectively. The content of *p*-coumaric acid was similar at 10 (1.26 mg/100 g DW) and 20 days (1.05 mg/100 g DW) of growth cycle (Table 3). 

The amount of rutoside was calculated as 3.06 and 23.24 mg/100 g DW after 10 and 20 days of growth period, respectively (Table 3). 

Quantitative analysis of the *N. officinale* herb extracts using the HPLC-DAD method showed high contents of protocatechuic acid (196.11 mg/100 g DW) and isoquercitrin (57.05 mg/100 g DW). The other phenolic acids were found to range from 3.10 (*p*-coumaric acid) to 26.55 mg/100 g DW (gallic acid). In the case of flavonoids, the content of rutoside was determined as 7.20 mg/100 g DW and kaempferol *O*-rhamnhexoside as 0.18 mg/100 g DW. The total content of flavonoids in the analyzed herb extract was 64.43 mg/100 g DW (Table 3).

### 2.5. Antioxidant Potential

In the antioxidant potential analysis, conducted using CUPRAC (CUPric reducing antioxidant activity), FRAP (Ferric Reducing Ability of Plasma), and DPPH (1,1-diphenyl-2-picrylhydrazyl) assays, a higher antioxidant potential was shown by the CUPRAC method for the biomass harvested after 10 days (3.19 mmol TE/100 g DW) compared with the cultures grown over 20 days (2.46 mmol TE/100 g DW) (Table 4). The antioxidant potential evaluated for the extracts from the *N. officinale* herb equaled 4.45 mmol TE/100 g DW (Table 4).

Similarly, in the FRAP analysis, a higher antioxidant potential was detected for microshoot extracts grown for 10 days (0.75 mmol TE/100 g DW) than for 20 days (0.67 mmol TE/100 g DW) (Table 4). In the case of extracts from *N. officinale* herb, the antioxidant potential was equal to 0.76 mmol TE/100 g DW (Table 4). 

In the DPPH analysis, stronger antioxidant potential was observed for the extracts from bioreactor cultures grown over 20 days (23.79 mmol TE/100 g DW) than the microshoots grown over 10 days (21.63 mmol TE/100 g DW) (Table 4). For the herb extract, the antioxidant potential was estimated to be 26.32 mmol TE/100 g DW (Table 4).

### 2.6. Anti-Inflammatory Activity

The anti-inflammatory activity was confirmed based on the in vitro inhibition of 15-lipoxygenase (15-LOX), cyclooxygenase-1 (COX-1), cyclooxygenase-2 (COX-2) and phospholipase A_2_ (sPLA_2_) enzymes. The extracts of *N. officinale* biomass maintained in vitro over 10 days in RITA^®^ bioreactors showed moderate inhibition of 15-LOX and very low inhibition of sPLA_2_. However, a relatively high inhibitory activity was observed against COX-1 and COX-2 enzymes (Table 5). 

The *N. officinale* bioreactor cultures inhibited 15-LOX at a level of 15.9% at 16.5 µg/mL. The most promising results were detected for in vitro inhibition against COX-1 and COX-2 enzymes. Between these two, the highest inhibition was noted against COX-2—75.4, 73.5, and 34.5% at 1.7, 16.5, and 165.0 µg/mL, respectively (Table 5). Evaluation of COX-1 inhibition showed that *N. officinale* bioreactor cultures inhibited this enzyme at a significant level of 40.5, 33.3, and 29.2% at a concentration of 16.5, 165.0, and 1.7 µg/mL, respectively (Table 5). In the case of sPLA_2_, the 3.8, 1.3, and 0.7% inhibition was observed for the in vitro extracts at 165.0, 16.5, and 1.7 µg/mL, respectively (Table 5).

Evaluation of 15-LOX inhibition by *N. officinale* herb extracts revealed that this enzyme was inhibited at 19.5% by the extract at a concentration of 16.5 µg/mL. The highest inhibition was noted for the COX-1 enzyme—42.6, 41.1, and 36.3% at 1.7, 165.0, and 16.5 µg/mL, respectively (Table 5). Evaluation of COX-2 inhibition showed that the herb extracts inhibited this enzyme at significant levels of 41.1, 39.8, and 33.0% at concentrations of 16.5, 165.0, and 1.7 µg/mL, respectively (Table 5). For the sPLA_2_ enzyme, the 5.3 and 2.1% inhibitions were observed at 1.7 and 165.0 µg/mL, respectively (Table 5).

### 2.7. Antimicrobial Activity

The *N. officinale* 10-days bioreactor microshoot cultures and herb extracts were tested for their antimicrobial properties with reference strains of bacteria and fungi presented in Table 6.

The minimal inhibitory concentration (MIC) determined for the extracts of in vitro *N. officinale* cultures ranged from 1.25 to 10 mg/mL (Table 6). For the tested Gram-positive bacterial strains (*S. aureus* and *S. epidermidis*), the MIC was equal to 5 mg/mL, while for Gram-negative bacteria, the values differed ranging from 1.25 (*H. pylori*) to 10 mg/L (*E. coli* and *P. aeruginosa*). The minimum bactericidal concentration (MBC) of the in vitro culture extracts was estimated at 1.25–20 mg/mL. Among the tested bacterial strains, the highest MBC (20 mg/mL) was noted for *S. aureus*, while the lowest (1.25 mg/L) was detected for *H. pylori.* The highest MBC/MIC ratio of 4 was obtained for *S. aureus*, whereas for other tested strains the ratio was lower than 4 (Table 6).

In the case of *N. officinale* herb extracts, the MIC for the tested bacterial strains ranged from 1.25 to 10 mg/mL (Table 6). For the Gram-positive bacterial strains, the MIC values were equal to 5 (*S. aureus*) and 10 mg/mL (*S. epidermidis*). For Gram-negative bacterial strains, the MIC values differed and ranged from 1.25 (*H. pylori*) to 10 mg/mL (*E. coli* and *P. aeruginosa*). The MBC values were calculated at 1.25–20 mg/mL. The highest MBC (20 mg/mL) was estimated for *S. aureus*, and the lowest (1.25 mg/mL) for *H. pylori.* As observed with biomass extracts, the highest MBC/MIC ratio of 4 was obtained for *S. aureus*, while for other strains the ratio was lower than 4 (Table 6).

For bioreactor microshoot culture extracts, the MIC for the tested fungal strains ranged from 1.25 to 20 mg/mL. The highest MIC (20 mg/mL) was determined for *C. parapsilosis*, and the lowest (1.25 mg/mL) for *T. menthagrophytes.* The highest minimum fungicidal concentration (MFC) (over 20 mg/mL) was noted for *A. niger* and *P. chrysogenum*, and the lowest (2.5 mg/mL) for *T. menthagrophytes.* The MFC/MIC ratio ranged from 1 to 4 for all tested strains (Table 6).

For *N. officinale* herb extracts, the MIC for fungal strains ranged from 2.5 to 10 mg/mL. The highest MIC (10 mg/mL) was calculated for *C. glabrata*, *C. parapsilosis*, *A. niger*, *P. chrysogenum*, and *M. canis*, while the lowest (2.5 mg/mL) was detected for *T. menthagrophytes.* The highest MFC (over 20 mg/mL) was estimated for *A. niger* and *P. chrysogenum*, whereas the lowest (2.5 mg/mL) was estimated for *T. menthagrophytes*. The MFC/MIC ratio ranged from 1 to 2 for all tested strains (Table 6). 

## 3. Discussion

This study is the first to establish the RITA^®^ bioreactor microshoot cultures of *N. officinale* involved the phytochemical studies and analysis of biological activities. We focused on the production of GSLs and two groups of polyphenols—phenolic acids and flavonoids. Biological activities, including antioxidant, anti-inflammatory, and antimicrobial properties, were tested for the first time in extracts obtained from *N. officinale* bioreactor microshoot cultures. The results obtained for these extracts were compared to those of the raw material.

A high amount of biomass was obtained from bioreactor microshoot cultures. The highest Gi factor of dried biomass (31.71) was recorded after 20 days of growth period. A comparison of these results with those of our former studies, which included *N. officinale* agar and agitated microshoot cultures [24], showed higher biomass increments in the studied RITA^®^ bioreactor cultures. In agitated microshoot cultures, the highest Gi (10.48) was 3.0-fold lower than in the bioreactor cultures. Comparing the highest Gi of bioreactor and agar cultures (5.05), a 6.3-fold increase was noted in bioreactor cultures than in agar cultures [24]. Jesionek et al. [26] studied biomass increases in *Ledum palustre* microshoot cultures in RITA^®^ bioreactors. After 28 days of in vitro culturing of *L. palustre*, the Gi of fresh weight was equal to 2.8, and in 20 days the Gi of *N. officinale* bioreactor cultures was 11.3-fold higher than that of *L. palustre* in vitro cultures [26]. For *Schisandra chinensis* in vitro microshoot cultures maintained in RITA^®^ bioreactors, the Gi factor was equal to 3.49 and 10.48 after 30 and 60 days, respectively [27]. The maximal Gi factor calculated for *N. officinale* bioreactor microshoot cultures was 9.1- and 3.0-fold higher than that of *S. chinensis* bioreactor cultures, respectively [27].

Analysis of the total GSLs contents indicated the differences between the *N. officinale* herb and bioreactor microshoot cultures (Table 1). In bioreactor microshoot cultures, the highest GSLs total content was detected after 10 days of growth period (261.97 mg/100 g DW), which was 3.0-fold lower than that of the *N. officinale* herb (799.47 mg/100 g DW). The studied material from the parent plant was whole herb (stems with leaves and flowers), while in vitro cultures are recognized as less morphologically differentiated (microshoot cultures). Former studies performed by Jeon et al. [15] showed that flowers of *N. officinale* contain higher content of GSLs in comparison to other herb organs. Moreover, in vitro cultures are known as systems that produce often lower levels of compounds compared to ground plants [28,29]. Nevertheless, in vitro culture systems distinguished by many qualities that support for their attractiveness as an object of promising scientific research [30].

In our previous studies [24], the highest total content of GSLs in the *N. officinale* agar and agitated cultures growing on MS medium containing 1 mg/L BA and 1 mg/L NAA was 164.97 and 182.80 mg/100 g DW, respectively. In *N. officinale* bioreactor microshoot cultures, the total GSLs content was 1.6-fold higher than in the agar cultures. The growth periods maintained were different—10 days for bioreactor cultures and 20 days for agar cultures, and, in turn, the Gi indexes also differed—16.54 and 3.79, respectively. Comparing the *N. officinale* agitated and bioreactor microshoot cultures, it was found that the total GSLs content was 1.4-fold higher in the bioreactor cultures than in the agitated cultures (Table 1) [24]. However, in both cultures, the maximal total content was observed after 10 days of growth period, while the Gi indexes was much higher for bioreactor cultures (16.54) than for agitated cultures (5.29) [24].

In another study, the total GSLs content in the *N. officinale* herb harvested at an interval of 1 month was estimated by spectrophotometry. The total content determined in our study was in compliance with the range of total GSLs found in this study at 592.21–1752.80 mg/100 g DW [31].

Our study shows that the GSLs contents differed in the extracts from bioreactor cultures and from the *N. officinale* herb. For methionine-derived GSLs (7-(methylsulfinyl)heptyl GSL and glucohirsutin), the content in herb extracts was significantly higher than in the extracts from bioreactor cultures. The main compound detected in both extracts was phenylalanine-derived GSL (gluconasturtiin); in the extracts from the herb, the content of gluconasturtiin was 3.5-fold higher than in the extracts from bioreactor cultures. In plant in vitro cultures, a 2.5-fold higher content of tryptophan-derived GSL (4-methoxyglucobrassicin) was found compared to the extracts from the herb. The content of another tryptophan-derived compound (glucobrassicin) was also significantly higher in the extracts from in vitro cultures than in the herb extracts (Table 2, Figure 2).

The qualitative and quantitative analysis of extracts from different organs of *N. officinale* (leaf, stem, root, flower, and seed) was performed by Jeon et al. using the HPLC-UV technique [15]. This team reported the presence of eight GSLs in the studied extracts: glucoiberin, glucotropaeolin, 4-hydroxyglucobrassicin, 7-(methylsulfinyl)heptyl GSL, glucohirsutin, glucobrassicin, 4-methoxyglucobrassicin, and gluconasturtiin. The authors found that gluconasturtiin was the main GSL present in the highest amount in flowers (7.39 mg/100 g DW). This content was 86.7-fold lower compared to our herb extracts and 24.8-fold lower than in the extracts obtained from in vitro cultures.

Extracts from *N. officinale* shoots were also studied by Giallourou et al. [16]. The identification and quantification of GSLs were performed using the LC-MS/MS technique. In the analyzed methanolic extracts, five GSLs were identified: gluconasturtiin, glucobrassicin, 4-hydroxyglucobrassicin, 4-methoxyglucobrassicin, and 7-(methylsulfinyl)heptyl GSL. The main compound detected in their study was gluconasturtiin at a content of 176.00 mg/100 g DW, which was 3.6-fold lower than that of our extracts from herb and was comparable to 10-day extracts of bioreactor microshoot cultures.

The *N. officinale* agar in vitro cultures were investigated by Rubin et al. [32]. This team reported two GSLs—gluconasturtiin and glucotropaeolin—in the methanolic extracts from the in vitro agar shoot cultures grown for 30 days on MS agar medium without plant growth regulators, containing 30 g/L sucrose and 4 g/L agar. The amount of gluconasturtiin (140.40 mg/100 g FW) determined in their study was 1.3-fold lower than in our bioreactor microshoot cultures harvested after 10 days of growth [32].

In our study, the higher total polyphenol content (3.74 mmol TE/100 g DW) was determined by the F-C assay for the microshoots cultured over 10 days, which was 1.4-fold higher in comparison to the herb extracts (Table 1). For the first time, studies on the total flavonoid pool in *N. officinale* in vitro cultures were performed in this study. The spectrophotometric analysis of the total flavonoid pool confirmed the greater contents of these compounds in the extracts from RITA^®^ bioreactor cultures grown over 10 days compared to those grown over 20 days, and these results were similar to the results obtained for herb extracts (Table 1).

The differences in the qualitative content of phenolic acids and flavonoids between the extracts from *N. officinale* bioreactor microshoot cultures and the *N. officinale* herb were confirmed using the UHPLC-DAD-ESI-MS method (Table 3). In the extracts of RITA^®^ bioreactor microshoot cultures, two phenolic acids (*p*-coumaric and ferulic acids) and one flavonoid (rutoside) were detected (Table 3). On the other hand, in the extracts of the herb, the presence of four phenolic acids (*p*-coumaric, gallic, ferulic and protocatechuic acids) and three flavonoids (isoquercitrin, kaempferol *O*-rhamnohexoside, and rutoside) was confirmed. Among flavonoids, only the presence of rutoside was confirmed in the extracts from bioreactor microshoot cultures. The maximal content of rutoside in these extracts (23.24 mg/100 g DW) was 3.2-fold higher than in the extracts from the herb (7.20 mg/100 g DW) (Table 3). For phenolic acids, they were not studied before in *N. officinale* in vitro cultures.

In the study performed by Zeb, a qualitative analysis of phenolic compounds in *N. officinale* leaf extracts was carried out [33], and the presence of caffeoylmalic acid, caftaric acid, coumaric acid derivative, *p*-coumaric acid, *p*-coumaric acid derivative, ferulic acid derivative, two gallic acid derivative, *p*-hydroxybenzoic acid, and sinapic acid was reported. In our extracts from the *N. officinale* herb, the presence of caffeoylmalic acid, caftaric acid, and *p*-hydroxybenzoic acid was not confirmed. Moreover, we detected protocatechuic acid as the main phenolic compound in herb extracts (Table 3). This author confirmed the presence of three flavonoids—apigenin, quercetin-3-(caffeoyldiglucoside)-7-glucoside, and kaempferol-3-(caffeoyldiglucoside)-7-rhamnoside—in the leaf extracts. However, in our study, we detected three different flavonoids—isoquercitrin, kaempferol *O*-rhamnohexoside, and rutoside—in the herb extracts (Table 3).

The young-baby leaves of *N. officinale* were analyzed in the study of Aires et al. [34]. Their evaluation of qualitative and quantitative contents of phenolic acids in the methanol:water (*v/v*) extracts confirmed the presence of four phenolic acids: caffeic, chlorogenic, dicaffeoyltartaric, and gallic acids. Similarly, in our study, we confirmed the presence of gallic acid and three another phenolic acids—*p*-coumaric, ferulic and protocatechuic acids—in herb extracts. The content of gallic acid (1.60 mg/100 g DW) determined in the study of Aires et al. was 16.6-fold lower in comparison to our results (Table 3). The authors also confirmed the presence of two flavonoids in their leaf extracts: isorhamnetin and rutoside. In our herb extracts, we detected the presence of rutoside, isoquercitrin, and kaempferol *O*-rhamnohexoside, among flavonoids (Table 3). The total content of analyzed polyphenols in our herb extracts was 1.5-fold lower than in the extracts from young-baby leaves [34].

The antioxidant potential of the studied RITA^®^ TIS-grown microshoot and herb extracts was analyzed using three assays: CUPRAC, FRAP, and DPPH.

In the CUPRAC assay, the antioxidant potential of herb extracts (4.45 mmol TE/100 g DW) was found to be 1.4-fold higher than in the extracts from RITA^®^ bioreactor cultures (3.19 mmol TE/100 g DW) (Table 4). In our former studies [24], we investigated the antioxidant potential using the CUPRAC assay for agar and agitated cultures growing on MS medium containing 1 mg/L BA and 1 mg/L NAA. For agar cultures, the highest antioxidant potential was confirmed after 20 days (3.26 mmol TE/100 g DW) and for agitated cultures after 10 days (2.65 mmol TE/100 g DW). The maximal antioxidant potential estimated for bioreactor cultures was similar to that of agar cultures and 1.2-fold higher than that of agitated cultures (Table 4) [24].

In the FRAP method, the maximal antioxidant potential (0.75 mmol TE/100 g DW) of bioreactor-grown cultures was found to be almost identical to that of the plant material (0.76 mmol TE/100 g DW) (Table 4). In previous studies [24], for the variant medium containing 1 mg/L BA and 1 mg/L NAA, the determination of antioxidant potential using the FRAP method showed higher potential for agar cultures (0.90 mmol TE/100 g DW) and lower for agitated cultures (0.55 mmol TE/100 g DW) after 10 days, which was, respectively, 1.2-fold higher and 1.4-fold lower in comparison with the bioreactor cultures studied in the present work (Table 4) [24].

The DPPH assay showed that the antioxidant potential reached the maximal value in RITA^®^ bioreactor cultures (23.79 mmol TE/100 g DW) and was 1.1-fold lower than in the herb extract (26.32 mmol TE/100 g DW) (Table 4). The antioxidant potential of *N. officinale* was also evaluated for agar and agitated cultures using the DPPH method in our previous studies [24]. For the variant medium containing 1 mg/L BA and 1 mg/L NAA, the DPPH method showed the highest antioxidant potential for agar cultures after 10 days (3.02 mmol TE/100 g DW) and agitated cultures after 20 days (19.57 mmol TE/100 g DW), which was 7.9- and 1.2-fold lower in comparison to the bioreactor cultures, respectively (Table 4) [24].

The anti-inflammatory assays confirmed the differences between bioreactor microshoots and herb extracts (Table 5). For 15-LOX, a 1.2-fold higher inhibition was observed for a similar concentration of plant extract compared to the RITA^®^ bioreactor microshoot extracts. In both extracts, the results were lower for NDGA used as a control (Table 5). For the COX-1 enzyme, the highest inhibition was shown by plant extracts at a concentration of 1.7 µg/mL, which was similar to the inhibition by bioreactor microshoot extracts achieved at a concentration of 16.5 µg/mL. In comparison to the control used for this enzyme (ibuprofen), the inhibition by both extracts was about 1.9-fold higher (Table 5). The highest differences were noted for the COX-2 enzyme; the strongest inhibition was observed with the bioreactor microshoot extracts, which was 1.8- and 3.6-fold higher than plant extracts and the control (ibuprofen), respectively (Table 5). For sPLA_2_, the inhibition by both extracts was lower. The highest inhibition was obtained for herb extracts, and it was almost 1.5-fold higher than bioreactor extracts and 17-fold lower in comparison to the control (thioetheramide-PC) (Table 5).

For the first time, we performed inhibition analysis for extracts from *N. officinale* herb and bioreactor microshoot cultures against 15-LOX, COX-1, COX-2, and sPLA_2_ enzymes. In the majority, the dose-dependent tendency of selected enzymes inhibition by plant extracts was observed. Nevertheless, inverse dose dependence can be noticed. It should be contributed to a wide confidence interval between dilutions, and thus the lack of significant difference. This can be seen for COX-1 inhibition by in vitro extract at 165 and 16.5 µg/mL, and by ex vitro plant material extract at a whole range of concentrations. However, the pronounced exceptions occurred for sPLA_2_ inhibition in the case of plant material and COX-2 in the case of in vitro culture extracts. Still, the inhibition activity of both extracts against sPLA2 is rather weak and thus of low significance, but this deviation from the competitive inhibition mechanism can also suggest the occurrence of inhibitor acceleration of the enzyme by active substances from ex vitro plant extract. The mechanism of inhibitor acceleration relies on allostery and multiple active sites [35]. Still, intriguing is the activity of diluted in vitro extract against COX-2. This result suggests the occurrence of selective COX-2 inhibitor accumulated in in vitro conditions. Our data let us suspect substances related to GSLs metabolism, which activity could be hypothetically suppressed by other co-accumulating secondary metabolites. Afterwards, the dilution activity of higher accumulating GSL-related intermediates is more pronounced. It is known that GSLs can be responsible for the inhibition of LOX- and COX-dependent inflammatory pathways. As reported by Herz et al. [35], isothiocyanates, the precursors of which are GSLs, block the COX and LOX pathway. Burčul et al. [36] showed that GSLs derived isothiocyanates are promising inhibitors of COX-2 enzymes. Almuhayawi et al. [37] showed results that increased accumulation of GSLs is linked with increased inhibition of LOX, COX-1, and COX-2 enzymes. This hypothesis should be confirmed in further studies. On the other hand, plant secondary metabolism is so rich and complicated that it could be concluded that particular properties are resultants of a few probably opposite activities. Thus, a parallel occurrence of components inhibiting as well as increasing the activity is possible due to the natural complexity of such a plant extract. Another study on the anti-inflammatory activity of *N. officinale* herb extracts focused on reduction in skin inflammation induced by croton oil via glucocorticoid receptor-dependent and NF-κB pathways in mice. The extract from *N. officinale* inhibited the croton oil-induced ear edema, reduced the infiltration of inflammatory cells and the levels of pro-inflammatory cytokines (MIP-2, IL-1β), and also inhibited the reduction of IkB-α protein expression induced by croton oil [38]. The study by Sadeghi et al. [18] also confirmed that oral intake of hydroalcoholic *N. officinale* herb extracts inhibited carrageenan-induced paw edema and formalin-evoked paw edema. Additionally, topical application of *N. officinale* reduced TPA (O-tetradecanoylphorbol-13-acetate)-induced ear edema, decreased swelling, and improved tissue damage [18].

To our best knowledge, this is the first study to analyze the antimicrobial activity of *N. officinale* biomass extracts from in vitro cultures. The antibacterial activity of *N. officinale* herb and bioreactor microshoot cultures against Gram-positive and Gram-negative bacteria was confirmed. Estimation of the MBC value allowed for establishing whether the extracts killed the bacteria or inhibited their growth. An antimicrobial agent is usually regarded as bactericidal if its MBC value is no more than four times the MIC value [39]. The MBC/MIC ratio determined for the extracts from bioreactor cultures was 2 and for the herb extracts was 1 (Table 6). Differences in the antifungal activity of extracts were observed for fungal strains, especially *C. albicans*, *C. parapsilosis*, *T. mentagrophytes*, and *M. canis*. The antimicrobial activity of extracts may result from the production of secondary metabolites which was also confirmed in the study. Moreover, the former studies [40] showed the antibacterial activity of extracts from *N. officinale* herb toward *E. coli*, *K. pneumoniae*, *E. faecalis*, and *B. cereus*. In the study of Quezada-Lazaro et al. [41], the antimicrobial activity of the herb extracts against *Mycobacterium tuberculosis* was confirmed. In the present study, we extended the analyses on these extracts using more fungal and bacterial strains for the first time.

## 4. Materials and Methods

### 4.1. Experimental In Vitro Cultures

Initial microshoot cultures of *N. officinale* were established and maintained as reported previously [25]. Experimental microshoots were cultivated in RITA^®^ TIS bioreactors (VITROPIC, Saint-Mathieu-de-Tréviers, France) (Figure 1 a,b) containing 200 mL of MS medium [42] with 3% (*w/v*) sucrose and supplemented with 1 mg/L BA and 1 mg/L NAA. The medium was identified as optimal for the cultivation of microshoots in our previous research [24]. The inoculum used in the present study was composed of 4 g microshoots. The nutrient media and biomass samples were collected after a growth period of 10 and 20 days (three series). After 30 days of growth period, the biomass died and was not tested further. The microshoots were grown under continuous exposure to an LED white light (2.75 W/m^2^) at a temperature of 25 ± 2 °C. The immersion cycle was set to 5 min every 1.5 h, at an aeration rate of 1.0 vvm.

### 4.2. Parent Plant Material

The *Nasturtium officinale* R. Br. plant material obtained from the Garden of Medicinal Plants, Faculty of Pharmacy, Jagiellonian University, Medical College, Cracow, Poland, loc. 50.01° N, 19.99° E, in May 2018 (Figure 1c). Voucher specimen (No. 102) has been deposited in the herbarium of this Garden. The material for analyses was lyophilized (Labconco Corporation, Kansas City, MO, USA) before.

### 4.3. In Vitro Biomass Increments

Biomass increments were measured by weighing biomass obtained after 10 and 20 days of growth period after lyophilization. For dry biomass, the growth index (Gi) was calculated according to the formula: Gi = $\frac{\left(Dw_{1}-Dw_{0}\right)}{Dw_{0}}\;$, where *Dw_1_* is the dry weight of microshoots observed at the end of the experiment and *Dw_0_* is the dry weight of the inoculum [4].

### 4.4. Analysis of GSL Contents Using UHPLC-DAD-MS/MS

#### 4.4.1. Materials and Reagents

Sinigrin was obtained from Sigma-Aldrich (St. Louis, MO, USA). Glucohesperin, glucohirsutin, gluconasturtiin, glucobrassicin, and 4-methoxyglucobrassicin were purchased from Phytoplan Diehm & Neuberger GmbH (Heidelberg, Germany). All other chemicals and reagents used in this study were of analytical grade.

#### 4.4.2. Isolation of dGSLs

Desulfoglucosinolates (dGSLs) were isolated from 100 mg of dried plant material as reported previously [43,44]. First, the plant material was extracted with methanol/H_2_O mixture (70:30, *v/v*; Gram-Mol d.o.o, Zagreb, Croatia). The resulting supernatant was loaded on a mini-column filled with DEAE-Sephadex A-25 anion-exchange resin (Sigma-Aldrich, St. Louis, MO, USA). The remaining nonpolar compounds were removed by washing the columns. To create appropriate conditions for the sulfatase reaction, the mini columns were washed once again with 20 mM NaOAc buffer (Merck, Darmstadt, Germany), followed by the addition of sulfatase (type H-1 from Helix pomatia; Sigma-Aldrich, St. Louis, MO, USA). The column was left overnight to allow the reaction to occur, and dGSLs were eluted the next day with ultrapure H_2_O (Merck Millipore, Burlington, MA, USA).

#### 4.4.3. UHPLC-DAD-MS/MS Analysis of dGSLs

This analysis was performed on a UHPLC-DAD-MS/MS apparatus (Ultimate 3000RS with TSQ Quantis MS/MS detector; Thermo Fischer Scientific, Waltham, MA, USA) using a Hypersil GOLD column (3.0 µm, 3.0 × 100 mm; Thermo Fischer Scientific, Waltham, MA, USA). A gradient consisting of solvent A (50 μM NaCl in H_2_O) and solvent B (acetonitrile:H_2_O, 30:70, *v/v*) was applied at a flow rate of 0.5 mL/min as follows: 0.14 min, 96% A and 4% B; 7.84 min, 14% A and 86% B; 8.96 min, 14% A and 86% B; 9.52 min, 5% A and 95% B; 13.16 min, 5% A and 95% B; 13.44 min, 96% A and 4% B; and 15.68 min, 96% A and 4% B. The column temperature was held at 25 °C, and the injection volume was 5 µL. The system was operated in the positive ion electrospray mode, with the electrospray interface H-ESI operating at a capillary voltage of 3.5 kV and a temperature of 350 °C. The signals were recorded at 227 nm by a DAD. dGSLs were quantified using an external calibration curve of pure desulfosinigrin (range from 13.56 to 542.50 µM). For each individual dGSL, response factors (RPFs) were taken into account in accordance with the literature as follows: RPF 1.0 for **1**, 1.1 for **3** [45], 0.95 for **4**, 0.29 for **5**, and 0.25 for **6** [46]; arbitrary 1.0 for **2**.

### 4.5. Total Phenolic Assay

Lyophilized (Labconco Corporation, Kansas City, MO, USA) and pulverized biomass obtained from in vitro cultures and from parent plant material was weighted (0.2 g) and extracted twice in 4 mL of methanol (STANLAB, Lublin, Poland) under sonication for 20 min (ultrasonic bath; POLSONIC 2, Warsaw, Poland). The extracts were centrifuged (7 min, 2000× *g*; MPW-223E; MPW, Warsaw, Poland) and filtered (0.22 μm syringe filters; Millex^®^ GP; Merck Millipore, Burlington, MA, USA). Total phenolic content was measured as described by Singelton et al. [47] with modifications reported by Bach et al. [48]. Water-diluted F-C reagent (5/2, *v/v*, 0.45 mL) was mixed with the studied extracts (100 μL). After 10 min, saturated Na_2_CO_3_ (0.45 mL) was added, and the samples were incubated for 2 h in dark at 25 °C. The samples were centrifuged and transferred to 96-well plates, and their absorbance was measured at 760 nm (Synergy II reader; Biotek, Winooski, VT, USA). The amounts of phenolic compounds were determined and expressed as TE in mmol/100 g DW. The analysis was done in triplicate (including reagents blank).

### 4.6. Total Flavonoid Assay

Total flavonoid content was estimated spectrophotometrically according to a method proposed by Ramos et al. [49]. Briefly, 100 μL of methanolic extract (prepared as described in Section 4.5) was mixed with 40 μL of 10% AlCl_3_ and 860 μL of 5% acetic acid in methanol. The samples were incubated for 20 min at room temperature and transferred to 96-well plates, and their absorbance was recorded at 425 nm (Synergy II, Biotek, Winooski, VT, USA). The amounts of flavonoids were determined and expressed as RE in mmol/100 g DW.

### 4.7. Qualitative Analysis of Phenolics by UHPLC-DAD-ESI-MS

This analysis was performed using an Ultimate 3000 series system (Dionex, Idstein, Germany), which was equipped with a dual low-pressure gradient pump with a vacuum degasser, an autosampler, a column compartment, and a DAD coupled with an Amazon SL ion-trap mass spectrometer (Bruker Daltonik GmbH, Bremen, Germany). Methanolic extract (prepared as described in Section 4.5) was used for the analysis. Compounds were separated from the analyzed extract with a Kinetex XB-C_18_ analytical column (100 × 2.1 mm × 1.7 µm; Phenomenex, Torrance, CA, USA). Temperature of the column was maintained at 25 °C. Elution was done with a mobile phase A (0.1% HCOOH in deionized water) and a mobile phase B (0.1% HCOOH in acetonitrile) in a multistep gradient as follows: 0 min, 1% B; 60 min, 26% B; and 90 min, 95% B. The flow rate was set to 0.300 mL/min during the analysis of the extracts and standards. Five microliters of each sample was introduced to the column using the autosampler. UV–Vis spectra were recorded at the wavelength range of 200–450 nm, and chromatograms were acquired at 254 and 325 nm. The eluate was introduced without splitting into the Amazon SL ion-trap mass spectrometer equipped with an ESI interface. The parameters for the ESI source were set as follows: nebulizer pressure, 40 psi; dry gas flow, 9 L/min; dry temperature, 300 °C; and capillary voltage, 4.5 kV. Analysis was carried out at a scan rate from *m/z* 70 to 2200. Compounds were analyzed in the negative ion mode, and MS^2^ fragmentations were performed using Smart Frag mode [50,51]. 

The compounds were identified based on the literature data and by comparing with chemical standards.

### 4.8. Analysis of Polyphenol Content by HPLC-DAD

This analysis was performed using the HPLC-DAD method described in previous works [52,53]. Methanolic extract (prepared as described in Section 4.5) was used. A HPLC-DAD system (Merck-Hitachi, Merck KGaA, Darmstadt, Germany) and a Purospher RP-18e analytical column (4 × 250 nm, 5 mL; Merck) were used for the analysis. Elution was done with a mobile phase A (methanol:0.5% acetic acid, 1:4, *v/v*) and a mobile phase B (methanol). The gradient program was set as follows: 0–20 min, 0% B; 20–35 min, 0–20% B; 35–45 min, 20–30% B; 45–55 min, 30–40% B; 55–60 min, 40–50% B; 60–65 min, 50–75% B; and 65–70 min, 75–100% B. The hold time was 15 min. The other parameters were as follows: temperature, 25 °C; flow rate, 1 mL/min; injection volume, 10 μL; and detection wavelength, 254 nm. Quantitative analysis was carried for the following compounds detected using UHPLC-DAD-ESI-MS: *p*-coumaric acid, ferulic acid, gallic acid protocatechuic acid and rutoside (all compounds were purchased from Sigma-Aldrich Co. St. Louis, MO, USA).

### 4.9. Antioxidant Capacity

#### 4.9.1. CUPRAC Assay of Total Antioxidant Capacity

The CUPRAC method described by Ozyurek et al. [54] and modified by Biesaga-Kościelniak et al. [55] was used to determine the total antioxidant activity in the extracts obtained from the tested biomass. For this, methanolic extracts of in vitro cultures and the parent plant herb (prepared as described in Section 4.5) were used. Briefly, 50 μL of the extracts was mixed with 50 μL aliquots of 10 mmol/L Cu^2+^, 7.5 mmol/L neocuproine, and 1 mol/L ammonia-acetate buffer (pH 7.0). Then, the samples were mixed and incubated for 15 min at 25 °C. Their absorbance was measured at 425 nm in 96-well plates (Synergy II, Biotek, Winooski, VT, USA). The antioxidant pool was determined and expressed as mmol TE/100 g DW. The measurements were performed in triplicate (including reagents blank).

#### 4.9.2. FRAP Assay

The antioxidant potential of the extracts obtained from the tested biomass was also estimated using the FRAP method [56]. This analysis was done in 96-well plates. Briefly, 10 mmol/L solution of TPTZ (2,4,6-tris(2-pyridyl)-s-triazine) in 40 mmol/L HCl was mixed with 20 mmol/L of FeCl_3_⋅6H_2_O and 300 mmol/L of pH 3.6 acetate buffer (1/1/10, *v/v/v*). Then, 50 µL of plant extract was mixed with 150 µL of the prepared chromophore. After incubation for 5 min, the absorbance of the sample was read at 593 nm (Synergy II, Biotek, Winooski, VT, USA). The measurements were done in triplicate (including reagents blank).

#### 4.9.3. DPPH Radical-Scavenging Activity Assay

The free radical-scavenging activity of the extracts was determined using the stable radical DPPH [57]. For this assay, plant extract (50 µL) was added to 150 µL of DPPH methanolic solution. The sample was mixed and incubated for 60 min, and then its absorbance was read at 517 nm (Synergy II, Biotek, Winooski, VT, USA). The measurements were performed in triplicate (including reagents blank).

### 4.10. Anti-Inflammatory Activity

The methanolic extracts of *N. officinale* herb and 10-days bioreactor RITA^®^ microshoot cultures of the plant were assayed for anti-inflammatory activity. Briefly, the plant extracts and in vitro extracts were serially diluted with methanol (concentrations: 165.0, 16.5, and 1.7 µg/mL). The anti-inflammatory activity of the extracts was tested based on the in vitro inhibition of 15-LOX, COX-1, COX-2, and sPLA_2_ enzymes. All the samples were measured in triplicate (100% enzyme activity, positive control inhibitor, plant extract and compound vehicle control samples).

The percent of inhibition was calculated according to the Equation (1):

(1) $$\%\mathrm{Inh}\;=\;(\frac{\mathrm{IA}\;-\;\mathrm{Inhibitor}}{\mathrm{IA}})\;\times \;100$$

where %Inh is the percent of inhibition and IA is 100% enzyme activity (without inhibitor), and Inhibitor represents the enzyme activity with inhibitor added.

#### 4.10.1. Inhibitory Activity Against 15-LOX

The inhibitory activity of the extracts against 15-LOX was tested using an assay kit (760700; Cayman Chem. Co., Ann Arbor, MI, USA) according to the manufacturer’s instructions. The assay involves the monitoring of hydroperoxides produced during lipooxygenation using purified soy 15-LOX with arachidonic acid as a substrate and 100 µmol/L nordihydroguaiaretic acid (NDGA) as a positive control inhibitor. The absorbance was measured in a 96-well plate (Synergy II, Biotek, Winooski, VT, USA) at 490 nm.

#### 4.10.2. Inhibitory Activity Against COX-1 and COX-2

The inhibitory activity of the extracts against COX-1 and COX-2 was tested using COX-1 (ovine) and COX-2 (human) inhibitor assay, with the peroxidase component of cyclooxygenase enzymes (701050; Cayman Chem. Co., Ann Arbor, MI, USA). The analysis was conducted according to the manufacturer’s instructions, with arachidonic acid (1.1 mmol/L) used as the substrate and 10 µmol/L ibuprofen as a positive control inhibitor. The increase in absorbance was monitored in a 96-well plate at 590 nm (Synergy II, Biotek, Winooski, VT, USA).

#### 4.10.3. Inhibitory Activity against sPLA_2_

The sPLA_2_ inhibitory activity of the extracts was tested using an assay kit (10004883; Cayman Chem. Co.) according to the manufacturer’s instructions. The method utilized human recombinant type V sPLA. Thiols released due to the cleavage of diheptanoyl thio-PC were measured kinetically using DTNB (Ellman’s reagent) at 420 nm (Synergy II, Biotek, Winooski, VT, USA). Thioetheramide-PC (1-palmitylthio-2-palmitoylamido-1,2-dideoxy-sn-glycero-3-phosphorylcholine) (100 µmol/L) was used as a positive control inhibitor.

### 4.11. Antimicrobial Activity

The extracts of 10-days bioreactor microshoot cultures and the *N. officinale* herb were also screened for antibacterial and antifungal activities using the microdilution broth method. The antimicrobial activity analysis was conducted according to the guidelines of the European Committee on Antimicrobial Susceptibility Testing (EUCAST) [58]. Mueller–Hinton broth and Mueller–Hinton broth with 5% lysed sheep blood were used for the growth of nonfastidious and fastidious bacteria, respectively, while RPMI (Roswell Park Memorial Institute Medium) with MOPS (3-(*N*-morpholino)propanesulfonic acid) was used for the growth of fungi as we described elsewhere [59,60]. The MIC of the tested derivatives was determined for a wide panel of reference microorganisms from the American Type Culture Collection (ATCC), including Gram-negative bacteria (*Escherichia coli* ATCC 25922, *Pseudomonas aeruginosa* ATCC 9027, *Helicobacter pylori* ATCC 43504), Gram-positive bacteria (*Staphylococcus aureus* ATCC 25923, *Staphylococcus aureus* ATCC 43300, *Staphylococcus epidermidis* ATCC 12228), and fungi (*Candida albicans* ATCC 10231, *Candida parapsilosis* ATCC 22019, *Candida glabrata* ATCC 90030, *Aspergillus niger* ATCC 16404, *Trichophyton mentagrophytes* ATCC 9533, *Penicillum chrysogenum* ATCC 10106, *Microsporum canis* clinical strain). Each experiment was done in triplicate, and representative data were presented.

## 5. Conclusions

This study analyzed, for the first time, the presence of GSLs, phenolic acids, and flavonoids in *N. officinale* microshoot cultures cultivated in TIS RITA^®^ bioreactors and evaluated the antioxidant, anti-inflammatory, and antimicrobial potential of the culture extracts. The results were compared with those obtained for the herb of the *N. officinale* parent plant.

In the bioreactor microshoot cultures, the Gi was very high and dependent on the duration of growth periods. The qualitative and quantitative analyses of GSLs and phenolic compounds confirmed the differences between the RITA^®^ TIS microshoot cultures and parent plant material.

The content of total GSLs, polyphenols, and flavonoids were found to be comparable or higher in the extracts from RITA^®^ bioreactor microshoot cultures than the parent plant herb.

Bioreactor microshoot cultures showed the lower production of the main GSLs (gluconasturtiin) and total GSLs but a higher amount of tryptophan-derived GSLs (glucobrassicin and 4-methoxyglucobrassicin), compared to parent plant extracts.

The antioxidant potential of the extracts from in vitro and in vivo plant materials was comparable. The highest accumulation of secondary metabolites and highest antioxidant activity were observed for extracts from 10-days bioreactor cultures. These extracts were also assessed for anti-inflammatory and antimicrobial activities, and it was found that the extracts from bioreactor microshoot cultures and parent plant material differed in the studied biological activities. We assume that the observed biological activities are mainly due to the GSL compounds, which were found as dominant in the studied extracts [17,18,19,20].

These results suggest the high potential of using RITA^®^ TIS for growing *N. officinale* microshoot cultures on a large scale. These cultures can be used as an alternative source of health-promoting compounds to parent plant material.

## Figures and Tables

**Table 1:** Total GSLs, polyphenols and flavonoids contents in *N. officinale* extracts from bioreactor microshoot cultures and the herb of the parent plant.

	In Vitro Cultures		Plant Material
	Growth Cycles (Days)		
	10	20	
Total glucosionolates (GSLs) (mg/100 g DW ± SD)	261.97 ± 21.14	113.70 ± 13.84	799.47 ± 4.89
Total polyphenols (mmol TE/100 g DW ± SD)	3.74 ± 0.25	3.10 ± 0.19	2.70 ± 0.31
Total flavonoids (mmol RE/100 g DW ± SD)	1.64 ± 0.08	0.95 ± 0.03	1.89 ± 0.20

Data expressed as the mean value ± SD (*n* = 3), TE—trolox equivalent, RE—rutoside equivalent.

**Table 2:** GSLs profile and quantitative content of detected compounds in *N. officinale* extracts from bioreactor microshoot cultures and the herb of the parent plant confirmed by UHPLC-DAD-MS/MS.

	No.	Glucosinolate (GSL) (Trivial Name)	*t*_R_ (min)	[M + Na]+	Content (mg/100 g DW ± SD)		
					In Vitro Cultures		Plant Material
					Growth Cycles (Days)		
					10 Days	20 Days	
*Met derived*	1	6-(Methylsulfinyl)hexyl GSL (Glucohesperin)	5.20	408	nd	tr	tr
	2	7-(Methylsulfinyl)heptyl GSL	6.20	422	tr	tr	92.27 ± 0.48
	3	8-(Methylsulfinyl)octyl GSL (Glucohirsutin)	7.20	436	tr	tr	42.79 ± 0.49
	4	7-(Methylsulfanyl)heptyl GSL	10.60	406	nd	nd	tr
	5	8-(Methylsulfanyl)octyl GSL	12.37	420	nd	nd	tr
*Phe derived*	6	2-Phenylethyl GSL (Gluconasturtiin)	7.70	366	182.93 ± 4.66	56.32 ± 7.62	640.94 ± 2.96
*Trp derived*	7	Indol-3-ylmethyl GSL (Glucobrassicin)	7.00	391	20.18 ± 4.04	7.62 ± 0.01	tr
	8	4-Methoxyindol-3-ylmethyl GSL (4-Methoxyglucobrassicin)	7.80	421	58.86 ± 12.44	49.76 ± 6.22	23.47 ± 0.96

[M + Na]^+^, sodium adduct of desulfoglucosinolate; DW, dry weight; tr < 0.1 μmol/g DW; nd, not detected. Data are expressed as the mean value ± SD (*n* = 3). *Met derived*—methionine-derived glucosinolates; *Phe derived*—phenylalanine-derived glucosinolates; *Trp derived*—tryptophan-derived glucosinolates.

**Table 3:** Phenolic profile and contents of detected compounds in *N. officinale* extracts from bioreactor microshoots cultures and from the herb of the parent plant confirmed by UHPLC-DAD-ESI-MS and HPLC-DAD.

No.	Compound	*t*_R_ (min)	UV-Vis	[M − H]^-^ *m/z*	MS^2^ Ions	Content (mg/100 g DW ± SD)		
						In Vitro Cultures		Plant Material
						Growth Cycles (Days)		
						10 Days	20 Days	
9	Gallic acid ^s^	3.4	269	169	125	nd	nd	26.55 ± 4.72
10	Protocatechuic acid ^s^	4.1	258, 287	152	-	nd	nd	196.11 ± 18.23
11	Undefined compound	9.7	260	426	-	nd	nd	nd
12	Undefined compound	15.0	253	283	-	nd	nd	nd
13	Undefined compound	16.2	250	337	-	nd	nd	nd
14	Protocatechuic acid derivative	20.4	258, 287	387	153	nd	nd	nd
15	Caffeic acid derivative	22.1	280, 323	325	163	nd	nd	nd
16	Undefined compound	22.9	264	417	207	nd	nd	nd
17	Phenolic acid derivative	24.8	295, 309	431	385, 223	nd	nd	nd
18	Caffeic acid derivative	25.3	297, 325	355	265, 217, 193	nd	nd	nd
19	Gluconasturtiin ^s^	28.1	220	422	342, 259, 229, 180	nd	nd	nd
20	Glucohirsutin ^t^	28.4	220	492	428, 275	nd	nd	nd
21	Phenolic acid derivative	28.8	300, 318	308	175	nd	nd	nd
22	Caffeic acid derivative	33.3	298, 323	363	319, 244, 161	nd	nd	nd
23	Caffeic acid derivative	35.3	300, 328	695	651, 489	nd	nd	nd
24	*p*-Coumaric acid ^s^	36.7	313	163	133	1.26 ± 0.02	1.05 ± 0.01	3.10 ± 0.21
25	Ferulic acid ^s^	39.9	260, 327	193	-	9.28 ± 0.87	1.66 ± 0.23	12.69 ± 1.13
26	Rutoside ^s^	41.3	overlaped	609	301	3.06 ± 0.28	23.24 ± 1.98	7.20 ± 0.67
27	Isoquercitrin ^s^	42.1	260, 351	463	301	nd	nd	57.05 ± 5.11
28	Kaempferol *O*-rhamnohexoside	45.6	260, 341	593	447, 285	nd	nd	0.18 ± 0.02
29	Sinapic acid derivative	51.3	264, 336	753	529, 289, 223	nd	nd	nd
30	Undefined compound	66.8	230	329	-	nd	nd	nd
31	Undefined compound	71.9	225	307	289, 235, 185	nd	nd	nd
32	Undefined compound	73.7	231	311	293, 181, 155	nd	nd	nd

^s^—authentic standard was used for comparison, ^t^—tentative assignment, nd-not detected. Data expressed as the mean value ± SD (*n* = 3).

**Table 4:** Antioxidant potential estimated by CUPRAC, FRAP and DPPH methods (expressed in mmol TE/100 g DW ± SD) in *N. officinale* extracts from bioreactor microshoot cultures and from the herb of the parent plant.

Method	In Vitro Cultures		Plant Material
	Growth Cycles (Days)		
	10 Days	20 Days	
CUPRAC	3.19 ± 0.20	2.46 ± 0.12	4.45 ± 0.02
FRAP	0.75 ± 0.05	0.67 ± 0.20	0.76 ± 0.08
DPPH	21.63 ± 2.77	23.79 ± 3.62	26.32 ± 8.23

CUPRAC—CUPric reducing antioxidant activity, FRAP—Ferric Reducing Ability of Plasma, DPPH—1,1-diphenyl-2-picrylhydrazyl assays; TE—trolox equivalent. Data expressed as the mean value ± SD (*n* = 3).

**Table 5:** In vitro inhibition activity (%Inh ± SD) of studied extracts from *N. officinale* microshoot cultures grown in RITA^®^ bioreactors over 10 days growth periods, and *N. officinale* herb extracts against 15-LOX, COX-1, COX-2 and sPLA_2_ enzymes.

Material		Concentrations (µg/mL)	%Inh ± SD						
			15-LOX	COX-1		COX-2		sPLA_2_	
In vitro cultures		165.0	no inhibition	33.3 ± 3.7		34.5 ± 3.8		3.8 ± 0.2	
		16.5	15.9 ± 1.1	40.5 ± 4.5		73.5 ± 8.1		1.3 ± 0.1	
		1.7	no inhibition	29.2 ± 3.2		75.4 ± 8.3		0.7 ± 0.1	
Plant material		165.0	no inhibition	41.1 ± 4.5		39.8 ± 4.4		2.1 ± 0.1	
		16.5	19.5 ± 1.4	36.3 ± 4.0		41.1 ± 4.5		no inhibition	
		1.7	no inhibition	42.6 ± 4.7		33.0 ± 3.6		5.3 ± 0.2	
Control	NDGA	30.2 (100 µM)	23.0 ± 2.0	-	-	-	-	-	-
	Ibuprofen	2.1 (10 µM)	-	-	23.0 ± 2.5	21.0 ± 2.0		-	-
	Thioetheramide-PC	73.6 (100 µM)	-	-	-	-	-	-	91.0 ± 4.0

15-LOX—15-lipoxygenase, COX-1 and -2, cyclooxygenase-1 and -2, sPLA_2_—phospholipase A_2_ enzymes; %Inh—percent of enzyme activity inhibition; SD—standard deviation (*n* = 3), NDGA—nordihydroguaiaretic acid; Thioetheramide-PC—1-palmitylthio-2-palmitoylamido-1,2-dideoxy-sn-glycero-3-phosphorylcholine.

**Table 6:** Antimicrobial activity of studied extracts from *N. officinale* microshoot cultures grown in RITA^®^ bioreactors over 10 days growth periods, and *N. officinale* herb extracts, against selected bacterial and fungi strains.

Microorganism		In Vitro Cultures			Plant Material		
		MIC (mg/mL)	MBC or MFC (mg/mL)	MBC/MIC or MFC/MIC Ratio	MIC (mg/mL)	MBC or MFC (mg/mL)	MBC/MIC or MFC/MIC Ratio
**Gram(+) bacteria**	*Staphylococcus aureus* ATCC 25923	5	10	2	5	10	2
	*Staphylococcus aureus* ATCC 43300	5	20	4	5	20	4
	*Staphylococcus epidermidis* ATCC12228	5	10	2	10	10	1
**Gram(-) bacteria**	*Escherichia coli* ATCC 25922	10	10	1	10	10	1
	*Pseudomonas aeruginosa* ATCC 9027	10	10	1	10	10	1
	*Helicobacter pylori* ATCC 43504	1.25	1.25	1	1.25	1.25	1
**Fungi**	*Candida albicans* ATCC 102231	10	20	2	5	10	2
	*Candida parapsilosis* ATCC 22019	20	20	1	10	20	2
	*Candida glabrata* ATCC 90030	10	10	1	10	10	1
	*Aspergillusniger* ATCC 16404	10	>20	nd	10	>20	nd
	*Penicillum chrysogenum* ATCC 10106	10	>20	nd	10	>20	nd
	*Trichophyton menthagrophytes* ATCC 9533	1.25	2.5	2	2.5	2.5	1
	*Microsporum canis* clinical strain	5	20	4	10	20	2

nd—not detected; MIC—minimal inhibitory concentration; MBC—minimum bactericidal concentration; MFC—minimum fungicidal concentration.

## Supplementary Materials

The following are available online, Figure S1: The chromatograms of the desulfoglucosinolates standards used, Figure S2: UV-Vis and MS^2^ spectra at 15V ionization of all desulfoglucosinolates detected. Numbers correspond to the Table 2.
