# Supplementary material for: Phytochemical and Biological Activity Studies on Nasturtium officinale (Watercress) Microshoot Cultures Grown in RITA® Temporary Immersion Systems
Source: Molecules. 2020 Nov 11;25(22):5257. doi: 10.3390/molecules25225257 (PMC7696031; doi:10.3390/molecules25225257)
Supplement: Supplementary file 1 [file molecules-25-05257-s001.pdf]

Supplementary Data

Phytochemical and Biological Activity Studies on *Nasturtium officinale* (Watercress) Microshoot Cultures Grown in RITA® Temporary Immersion Systems

Marta Klimek-Szczykutowicz[1], Michał Dziurka[2], Ivica Blažević[3], Azra Đulović[3], Sebastian Granica[4], Izabela Korona-Głowniak[5], Halina Ekiert[1], Agnieszka Szopa[1]*

[1] Chair and Department of Pharmaceutical Botany, Faculty of Pharmacy, Jagiellonian University, Medical College, Medyczna 9, 30-688 Kraków, Poland, marta.klimek-szczykutowicz@doctoral.uj.edu.pl (M.K.-S.), a.szopa@uj.edu.pl (A.S.), mfekiert@cyf-kr.edu.pl (H.E.)

[2] Polish Academy of Sciences, The Franciszek Górski Institute of Plant Physiology, Niezapominajek 21, 30-239 Kraków, Poland, email: m.dziurka@ifr-pan.edu.pl (M.D.)

[3] Department of Organic Chemistry, Faculty of Chemistry and Technology, University of Split, Ruđera Boškovića 35, 21000 Split, Croatia, e-mail: blazevic@ktf-split.hr (I.B.); azra@ktf-split.hr (A.Đ.)

[4] Department of Pharmacognosy and Molecular Basis and Phytotherapy, Medical University of Warsaw, Banacha 1, 02-097 Warszawa, Poland, e-mail: sgranica@wum.edu.pl (S.G.)

[5] Department of Pharmaceutical Microbiology, Faculty of Pharmacy with Division of Medical Analytics, Medical University of Lublin, Chodźki 1, 20-093 Lublin, Poland, e-mail: iza.glowniak@umlub.pl (I.K.-G.)

*Correspondence: a.szopa@uj.edu.pl; Tel.: +48-12-620-5436 (A.S.)

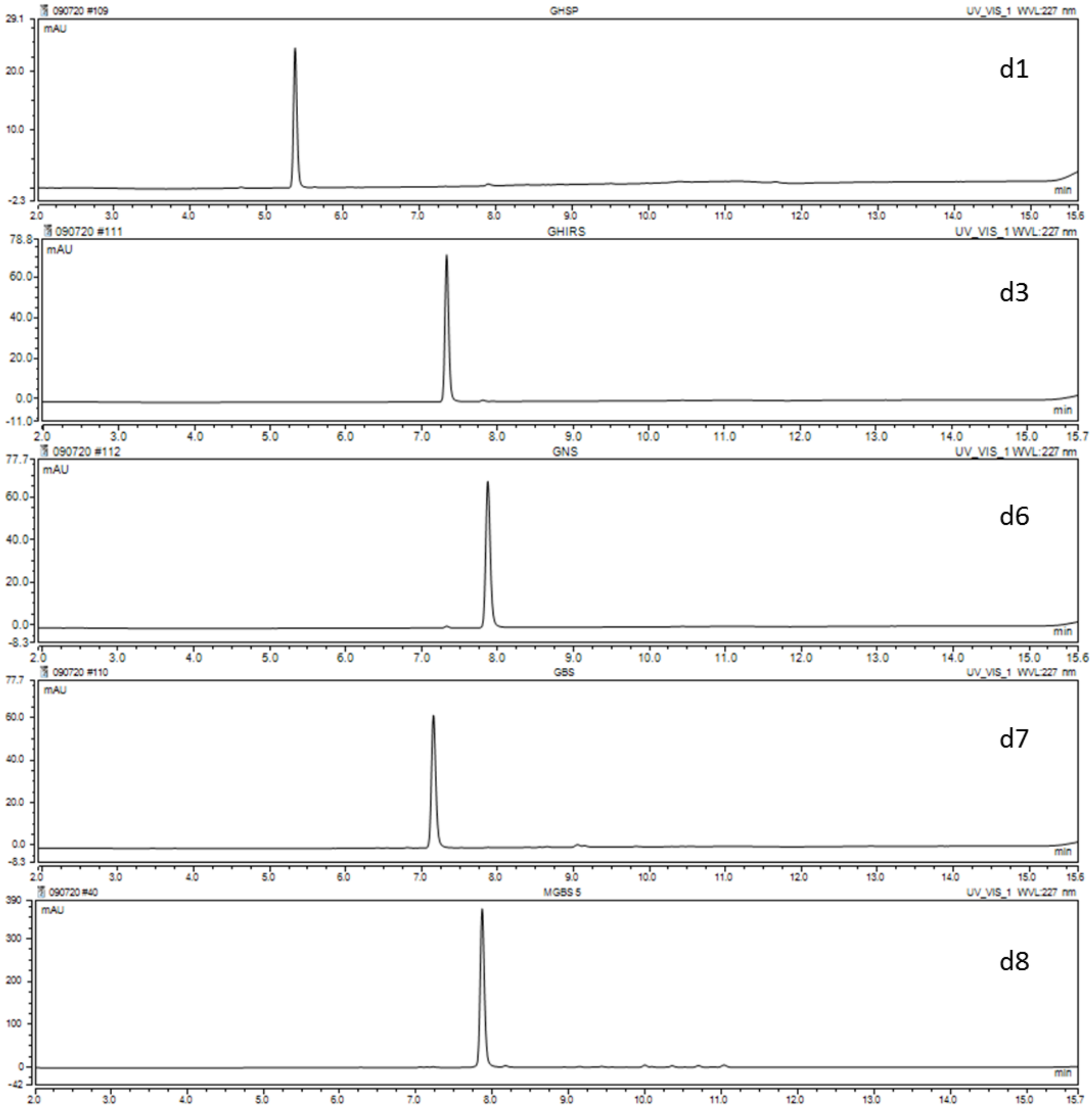

Figure S1. The chromatograms of the desulfoglucosinolates standards used.

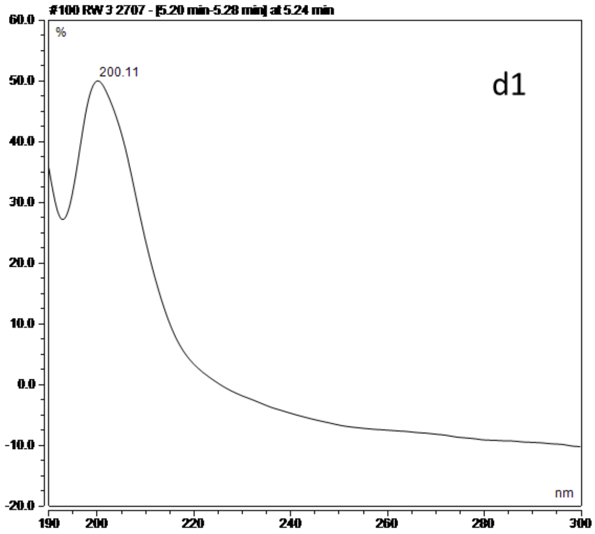
#100 RW 3 2707 - [5.20 min-5.28 min] at 5.24 min
d1
200.11
%
nm

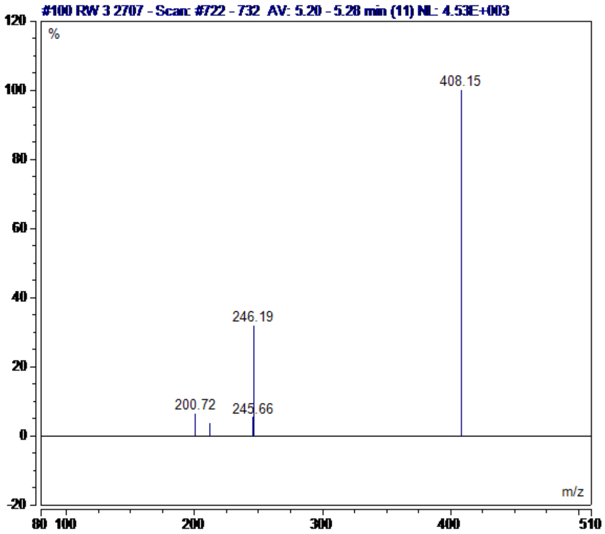
#100 RW 3 2707 - Scan: #722 - 732 AV: 5.20 - 5.28 min (11) NL: 4.53E+003
408.15
246.19
200.72
245.66
%
m/z

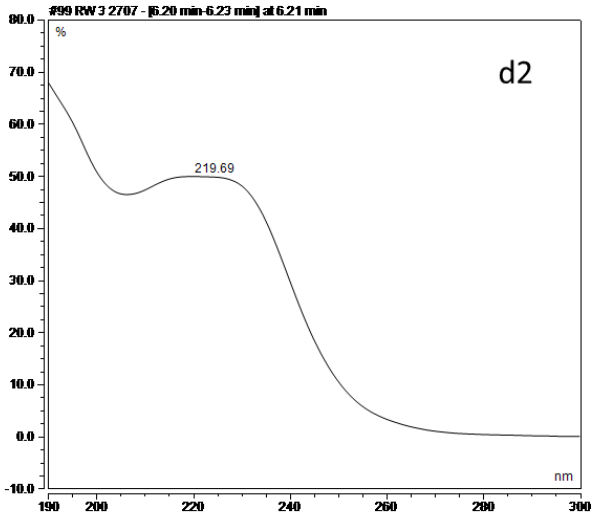
#99 RW 3 2707 - [6.20 min-6.23 min] at 6.21 min
d2
219.69
%
nm

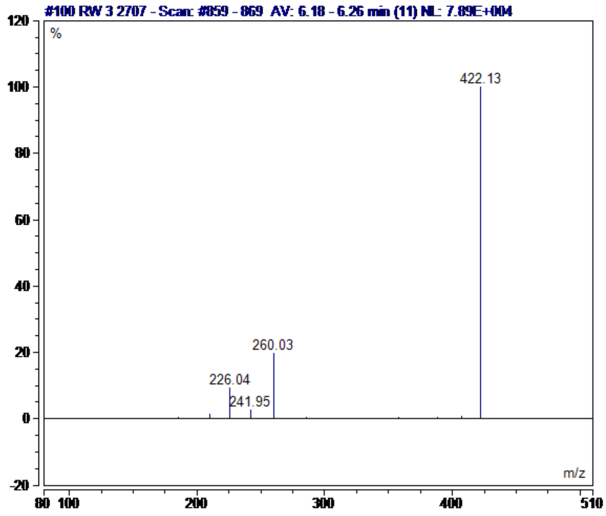
#100 RW 3 2707 - Scan: #859 - 869 AV: 6.18 - 6.26 min (11) NL: 7.89E+004
422.13
260.03
226.04
241.95
%
m/z

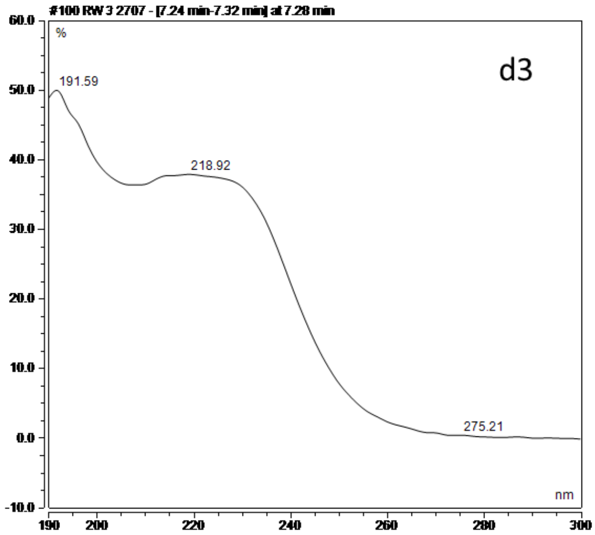
#100 RW 3 2707 - [7.24 min-7.32 min] at 7.28 min
d3
191.59
218.92
275.21
%
nm

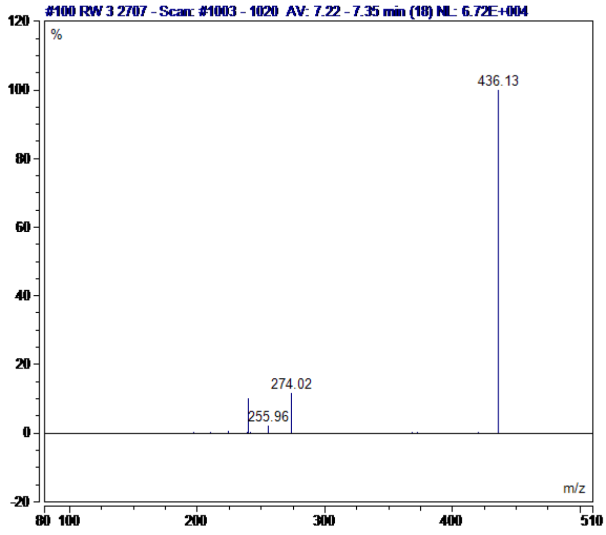
#100 RW 3 2707 - Scan: #1003 - 1020 AV: 7.22 - 7.35 min (18) NL: 6.72E+004
436.13
274.02
255.96
%
m/z

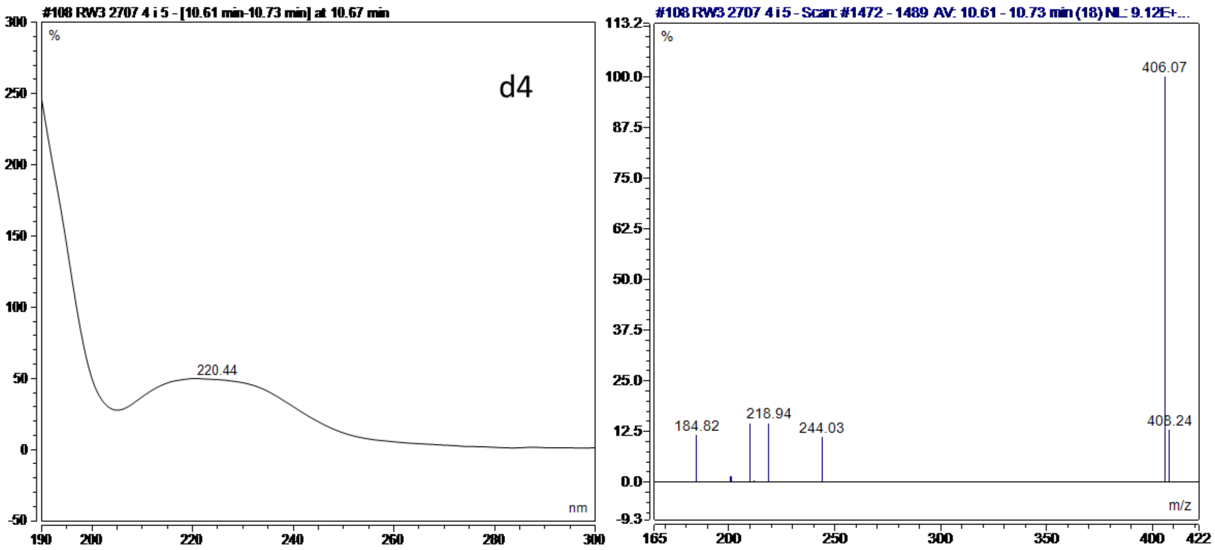
#108 RW3 2707 4 i 5 - [10.61 min-10.73 min] at 10.67 min
d4
220.44
nm
#108 RW3 2707 4 i 5 - Scan: #1472 - 1489 AV: 10.61 - 10.73 min (18) NL: 9.12E+...
406.07
184.82
218.94
244.03
m/z

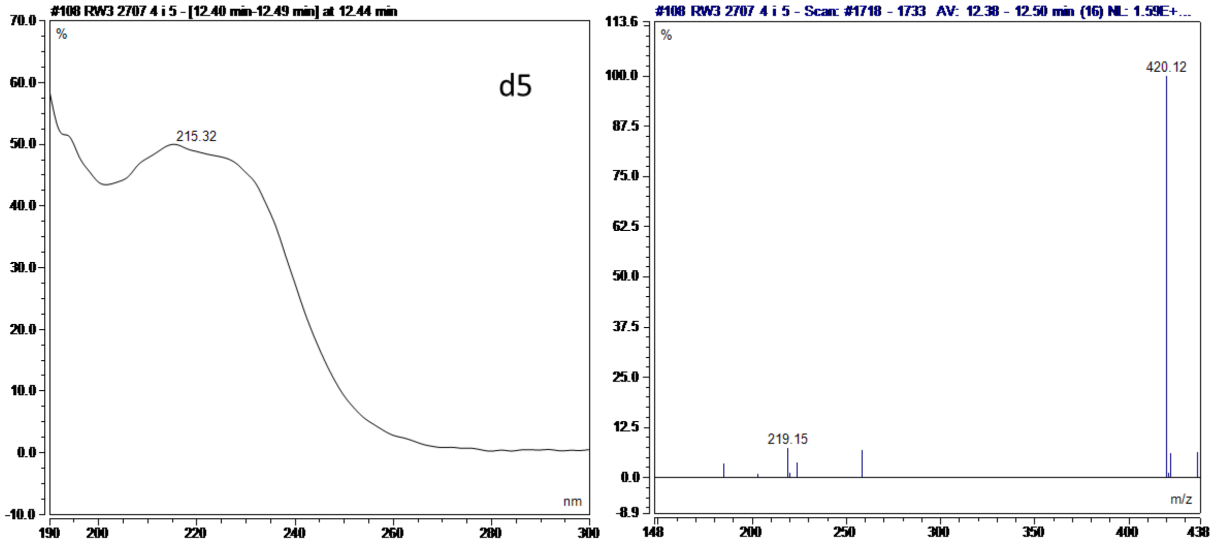
#108 RW3 2707 4 i 5 - [12.40 min-12.49 min] at 12.44 min
d5
215.32
nm
#108 RW3 2707 4 i 5 - Scan: #1718 - 1733 AV: 12.38 - 12.50 min (16) NL: 1.59E+...
420.12
219.15
m/z

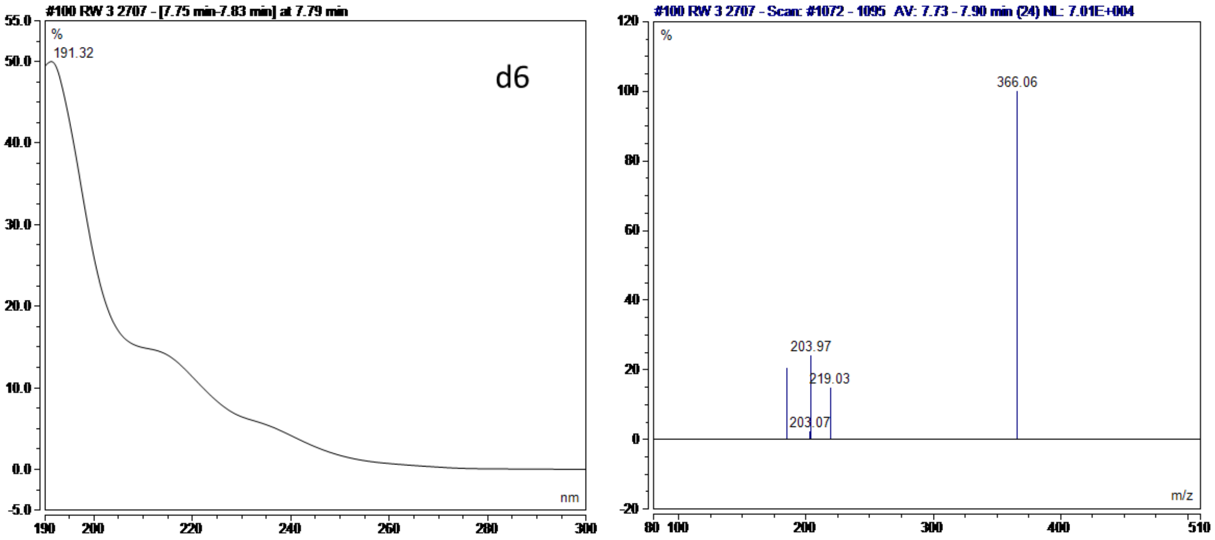
#100 RW 3 2707 - [7.75 min-7.83 min] at 7.79 min
d6
191.32
nm
#100 RW 3 2707 - Scan: #1072 - 1095 AV: 7.73 - 7.90 min (24) NL: 7.01E+004
366.06
203.97
219.03
203.07
m/z

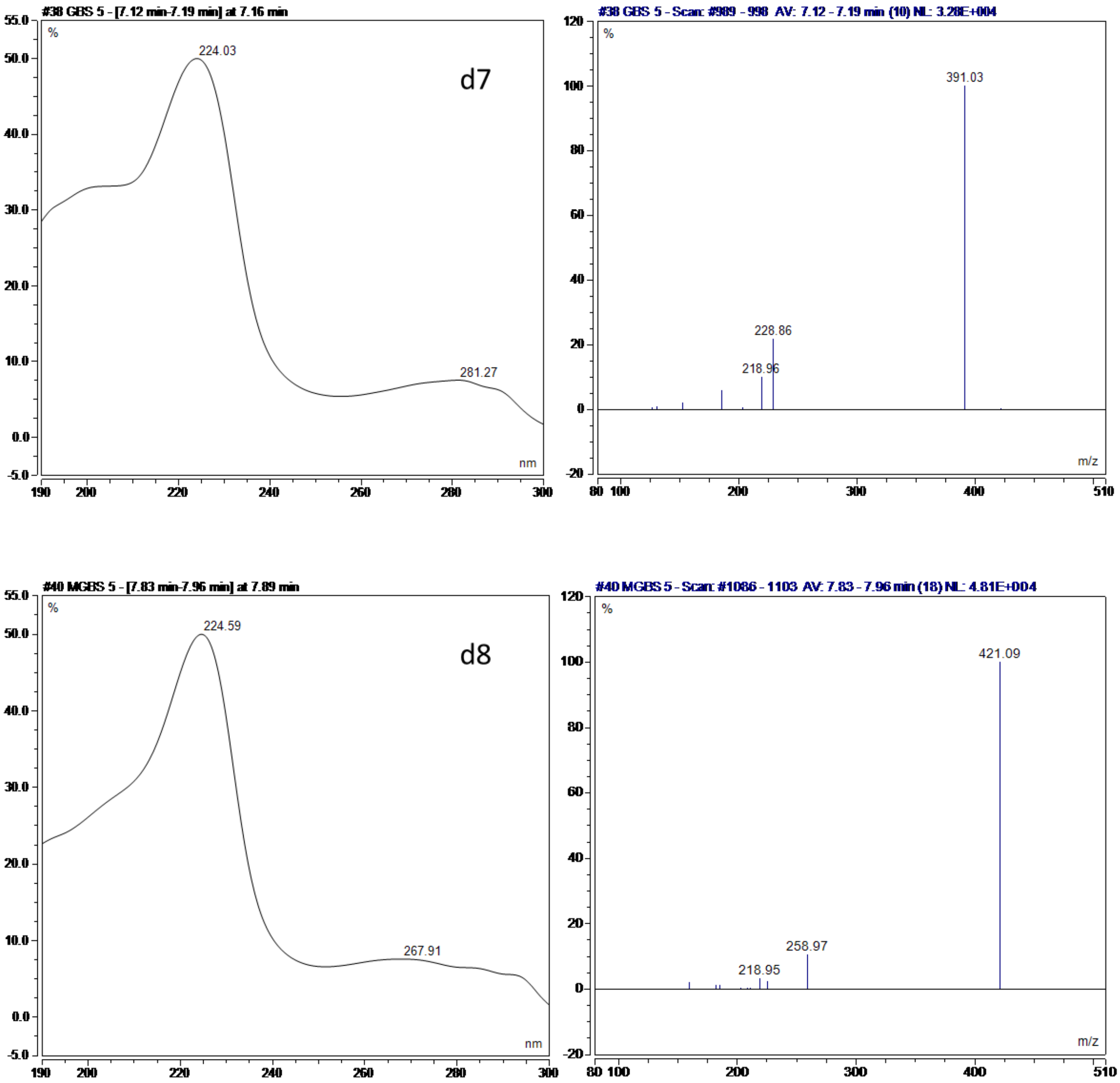

Figure S2. UV-Vis and MS^2 spectra at 15V ionization of all desulfoglucosinolates detected. Numbers correspond to the Table 2.
